# Novel risk scoring system for patients with metastatic castration-resistant prostate cancer treated with lutetium-177–PSMA-617

**DOI:** 10.1093/oncolo/oyag269

**Published:** 2026-07-11

**Authors:** Margo B Gerke, Angelo Marra, Michelle Jayaraj, Ahmet Yildrim, Akshay Bedmutha, Jacqueline T Brown, Bassel Nazha, Jacob E Berchuck, Ravi Bharat Parikh, Shahid Ahmed, Jordan Alana Ciuro, Caitlin Hartman, Greta Russler McClintock, Sarah Caulfield, Omer Kucuk, Bradley Curtis Carthon, David M Schuster, Saima Muzahir, Yuan Liu, Mehmet Asim Bilen

**Affiliations:** Emory University School of Medicine, Atlanta, GA 30322, United States; Biostatistics Shared Resource, Winship Cancer Institute, Emory University, Atlanta, GA 30322, United States; Emory University School of Medicine, Atlanta, GA 30322, United States; Emory University School of Medicine, Atlanta, GA 30322, United States; Department of Radiology and Imaging Sciences, Division of Nuclear Medicine and Molecular Imaging, Emory University, Atlanta, GA 30322, United States; Department of Hematology and Medical Oncology, Emory University School of Medicine, Atlanta, GA 30322, United States; Winship Cancer Institute of Emory University, Atlanta, GA 30322, United States; Winship Cancer Institute of Emory University, Atlanta, GA 30322, United States; Department of Hematology and Medical Oncology, Emory University School of Medicine, Atlanta, GA 30322, United States; Winship Cancer Institute of Emory University, Atlanta, GA 30322, United States; Department of Hematology and Medical Oncology, Emory University School of Medicine, Atlanta, GA 30322, United States; Winship Cancer Institute of Emory University, Atlanta, GA 30322, United States; Department of Hematology and Medical Oncology, Emory University School of Medicine, Atlanta, GA 30322, United States; Winship Cancer Institute of Emory University, Atlanta, GA 30322, United States; Department of Hematology and Medical Oncology, Emory University School of Medicine, Atlanta, GA 30322, United States; Winship Cancer Institute of Emory University, Atlanta, GA 30322, United States; Department of Hematology and Medical Oncology, Emory University School of Medicine, Atlanta, GA 30322, United States; Winship Cancer Institute of Emory University, Atlanta, GA 30322, United States; Department of Hematology and Medical Oncology, Emory University School of Medicine, Atlanta, GA 30322, United States; Winship Cancer Institute of Emory University, Atlanta, GA 30322, United States; Department of Hematology and Medical Oncology, Emory University School of Medicine, Atlanta, GA 30322, United States; Winship Cancer Institute of Emory University, Atlanta, GA 30322, United States; Department of Hematology and Medical Oncology, Emory University School of Medicine, Atlanta, GA 30322, United States; Winship Cancer Institute of Emory University, Atlanta, GA 30322, United States; Department of Hematology and Medical Oncology, Emory University School of Medicine, Atlanta, GA 30322, United States; Winship Cancer Institute of Emory University, Atlanta, GA 30322, United States; Department of Radiology and Imaging Sciences, Division of Nuclear Medicine and Molecular Imaging, Emory University, Atlanta, GA 30322, United States; Department of Radiology and Imaging Sciences, Division of Nuclear Medicine and Molecular Imaging, Emory University, Atlanta, GA 30322, United States; Department of Biostatistics and Bioinformatics, Emory University, Atlanta, GA 30322, United States; Emory University School of Medicine, Atlanta, GA 30322, United States; Department of Hematology and Medical Oncology, Emory University School of Medicine, Atlanta, GA 30322, United States; Winship Cancer Institute of Emory University, Atlanta, GA 30322, United States

**Keywords:** biomarker, radioligand therapy, metastatic castration-resistant prostate cancer, theranostics, lutetium-177–PSMA-617, risk scoring system, inflammation, body composition

## Abstract

**Purpose:**

Lutetium-177(^177^Lu)–PSMA-617 improves survival for patients with metastatic castration-resistant prostate cancer, but outcomes are heterogeneous and standardized risk stratification tools are lacking. We developed a novel risk stratification tool for patients treated with ^177^Lu–PSMA-617.

**Methods:**

We conducted a retrospective analysis of 163 patients at Emory Winship Cancer Institute. Four prognostic models using Cox proportional hazards models were constructed using best-subset variable selection and integrated into a composite model.

**Results:**

Among the 163 patients (52% White, 44% Black; 80% previous taxane use) body mass index decline during treatment was associated with shorter progression-free survival (PFS) and overall survival (OS) (HR = 1.75, *P* = .03; HR = 2.35, *P* = .031). Higher subcutaneous adipose tissue index (SATI)/skeletal muscle index (SMI) was independently associated with improved OS (HR = 0.5, *P* = .019), whereas higher visceral adipose tissue index (VATI)/SATI was independently associated with worse OS (HR = 2.62, *P* = .005). A body composition-based prognostic score identified VATI/SATI, SMI, and total adiposity as the strongest OS predictors (C = 0.629). The composite score incorporating inflammatory biomarkers and body composition demonstrated good discrimination (C-index = 0.732) and effectively stratified patients by PFS and OS. For PFS, relative risks across increasing risk quartiles were 0.34, 0.40, and 0.62 in compared with the highest risk quartile (*P* = .034). For OS, corresponding hazard ratios were 0.15, 0.19, and 0.54 (*P* = .003).

**Conclusions:**

A risk score incorporating body composition and inflammatory biomarkers demonstrates prognostic value for patients treated with ^177^Lu–PSMA-617.

Implications for Practice There are currently no widely adopted risk stratification tools for patients with metastatic castration-resistant prostate cancer treated with lutetium-177–PSMA-617. We developed a composite prognostic score integrating routinely available blood-based biomarkers and body composition metrics. The final prognostic score for overall survival included the systemic immune-inflammation index, the ratio of visceral adipose tissue to subcutaneous adipose tissue, Eastern Cooperative Oncology Group Performance Status, baseline hemoglobin, platelet-to-lymphocyte ratio, and neutrophil-to-eosinophil ratio. By using readily available clinical data, the composite score has potential for broad clinical applicability. It may assist oncologists in guiding risk stratification discussions with patients with metastatic castration-resistant prostate cancer being considering for treatment with lutetium-177–PSMA-617.

## Introduction

The prognosis for patients with metastatic castration-resistant prostate cancer (mCRPC) remains poor despite therapeutic advances. Lutetium-177(^177^Lu)–PSMA-617 has demonstrated survival benefits for patients with mCRPC who have progressed on androgen receptor pathway inhibitors (ARPIs) and is increasingly considered prior to taxane chemotherapy. Given the heterogeneity in outcomes and the increasing use of ^177^Lu–PSMA-617 prior to taxane chemotherapy, improved risk stratification is needed to guide patient treatment selection and sequencing.

Currently, eligibility for ^177^Lu–PSMA-617 is defined by clinical and imaging criteria; however, no widely accepted risk stratification system exists for patients who meet these eligibility criteria. Current imaging guidelines require at least 1 prostate-specific membrane antigen (PSMA)-positive metastatic lesion, defined as uptake greater than liver background without the presence of PSMA-negative lesions.^1^ Clinical exclusion criteria include life expectancy of less than 3 months, Eastern Cooperative Oncology Group (ECOG) performance status ≥3, or significant myelosuppression.^2^ While these criteria identify candidates for treatment, they do not distinguish patients who are most likely to benefit from ^177^Lu–PSMA-617.

Prognostic variables, including demographic factors, blood-based biomarkers, and body composition metrics may improve risk stratification for patients considering treatment with ^177^Lu–PSMA-617.^3–6^ All patients considered for treatment with ^177^Lu-PSMA-617 undergo a PSMA positron emission tomography (PET)/computed tomography (CT), which provides a reliable opportunity to extract prognostic radiographic metrics. Previous work has demonstrated that inflammatory biomarkers are associated with worse outcomes in patients with mCRPC treated with ^177^Lu-PSMA-617.^7^ These inflammatory markers correlate positively with muscle wasting and increased visceral adiposity, whereas subcutaneous adipose tissue shows an inverse trend.^7–11^ Visceral adiposity has specifically been associated with increased inflammation; in prostate cancer, periprostatic adipose tissue may promote a pro-inflammatory and pro-tumor environment through cytokine signaling, including secretion of IL-6.^12^^,^^13^

To date, there are no prognostic models incorporating blood-based systemic inflammatory markers and body composition metrics in patients treated with ^177^Lu–PSMA-617. In the present study, we investigated whether CT-derived body composition markers are prognostic for patients with mCRPC. Building on our previous work focused on inflammatory markers, we incorporated body composition metrics into a novel prognostic model to improve risk stratification for patients considering treatment with ^177^Lu–PSMA-617.

## Methods

### Patients and data collection

A total of 163 patients with mCRPC who were treated with ^177^Lu–PSMA-617 at Emory Winship Cancer Institute between July 10, 2020, and June 17, 2025, were retrospectively analyzed. The Emory Institutional Review Board approved the study and granted a waiver of informed consent. Patients who had received at least 1 cycle of ^177^Lu–PSMA-617 and met the PSMA PET/CT imaging criteria outlined in the VISION trial were included in the analysis.^1^ Information collected from retrospective chart review included PSA, neutrophils, lymphocytes, monocytes, eosinophils, alkaline phosphatase (ALP), hemoglobin, and platelets at baseline and at each subsequent ^177^Lu–PSMA-617 cycle. High-volume disease was defined as in the CHAARTED trial as 4 or more bone metastases, with at least 1 in the appendicular skeleton or visceral metastasis. Adverse events were documented using the Common Terminology Criteria for Adverse Events (CTCAE) definitions.^14^ An axial slice of the mid-section of the third lumbar vertebra was obtained from the CT portion of the PSMA-PET/CT obtained prior to treatment with ^177^Lu–PSMA-617 using Slice-O-Matic software (version 5.0, TomoVision). Body composition metrics were all collected pretreatment and included psoas muscle index (PMI), skeletal muscle index (SMI), visceral adipose tissue index (VATI), subcutaneous adipose tissue index (SATI), total adipose tissue index (TATI), visceral fat-to-subcutaneous fat ratio (VATI/SATI), and subcutaneous adipose tissue-to-skeletal muscle index (SATI/SMI), intermuscular fat index (IFI), and skeletal muscle attenuation (mean Hounsfield units). Body mass index (BMI) was collected before the initiation of ^177^Lu–PSMA-617 and at the time point of follow-up closest to completion of the final cycle of ^177^Lu–PSMA-617 that the patient received. Skeletal muscle area at the level of the third vertebra included the rectus abdominis, external and internal obliques, transversus abdominis, erector spinae, quadratus lumborum, and psoas muscles measured in square centimeters. Body composition metrics were collected by 1 trained author (M.B.G.). SMI, PMI, SATI, VATI, and TATI were divided by patients’ height in meters squared to obtain index values.^15^ Validated standard Hounsfield unit reference ranges were used for skeletal muscle (−29 to +150 HU), subcutaneous and intermuscular fat (−190 to −30 HU), and visceral fat (−150 to −50 HU).^15–17^ Blood-based systemic markers of inflammation were collected at the time point closest to initiation of ^177^Lu–PSMA-617 and are extensively detailed in prior work.^7^ Neutrophil-to-lymphocyte ratio (NLR) was calculated as absolute neutrophil count/lymphocyte count; monocyte-to-lymphocyte ratio (MLR) was calculated as monocyte count/lymphocyte count; platelet-to-lymphocyte ratio (PLR) was calculated as platelets/lymphocyte count; neutrophil-to-eosinophil ratio (NER) was calculated as absolute neutrophil count/absolute eosinophil count; pan-immune-inflammation value (PIV) was calculated as (absolute neutrophil count×platelets×monocytes)/lymphocytes, systemic immune-inflammation index (SII) was calculated as (neutrophils×platelets)/lymphocytes; hemoglobin-to-platelet ratio (HPR) was calculated as hemoglobin/platelets.^7^

### Statistical analysis

SAS version 9.4 and SAS macros were used for biostatistical analyses.^18^ Overall survival (OS) was the primary clinical endpoint assessed. Progression-free survival (PFS) and PSA50 response were secondary endpoint. OS was continuously evaluated as months from initiation of ^177^Lu–PSMA-617 to death from any cause with censoring at the last follow-up. PFS was continuously assessed and defined as PSA progression, imaging-based progression, or death, according to the Prostate Cancer Clinical Trials Working Group 3 (PCWG3) guidelines.^19^ PSA50 response was defined as a >50% decline in PSA since initiation of ^177^Lu–PSMA-617 and categorically assessed. Optimal dichotomous thresholds for inflammatory biomarkers and body composition metrics were identified using a bias-corrected log-rank test-based search algorithm to identify cut points that maximized OS discrimination.

To develop the composite risk scores, 4 separate Cox proportional hazards models were constructed to incorporate distinct prognostic domains: baseline patient characteristics, body composition metrics, inflammatory biomarker values, and a cumulative final model comprising candidate variables selected from all domain-specific models. Models were selected based on Harrell’s C-index, which reflects the model’s ability to correctly rank patients by risk. A composite risk score was derived for each domain from the β coefficients of each model, with each variable weighted proportionally to its coefficient. Variables were selected based on their contribution to overall model performance (C-index). Model performance was compared using the compareC R package to assess for differences in C-indices. The compareC package implements the nonparametric approach proposed by Kang et al for comparing correlated C-indices derived from survival data.^20^ This method estimates the two C-indices, their difference, and the variance of the difference while accounting for the correlation between models and then computes a corresponding 2-sided *P* value from the normal standard distribution. The risk score prognostication was assessed by stratifying the cohort into quartiles based on the risk score and comparing OS and PFS using multivariable analysis, Kaplan–Meier curves, and log-rank tests. Missing data were resolved using multiple imputation before to model development, and the proportional hazards assumption was assessed via Schoenfeld residuals.

## Results

### Patient and disease characteristics

Baseline demographics and body composition metrics of the cohort of 163 patients are displayed in Table 1. The median patient age was 73 years (interquartile range [IQR], 65-80). Fifty-two percent of patients identified as White, 43% identified as Black or African American, and the remainder identified as Asian, Hispanic, or declined to report. The median number of prior lines of therapy was 4 (IQR, 3-6), with all patients receiving prior ARPIs and approximately 80% having prior exposure to taxane-based chemotherapy. Patients received a median of 4 cycles of ^177^Lu–PSMA-617 (IQR, 2-6). The median baseline PSA was 68.5 ng/mL (IQR, 20.44-342, reference range, ≤4 ng/mL). The median BMI at the initiation of ^177^Lu–PSMA-617 treatment was 26.47 kg/m^2^ (IQR, 23.43-30.88). The median SMI was 41.21 (IQR, 36.27-47.38).

**Table 1.** Baseline patient demographics.

Variable	Total *N* = 163
**Patient race (%)**	
**White**	80 (51.61)
**Black**	66 (42.58)
**Asian**	6 (3.87)
**Hispanic**	3 (1.94)
**CHAARTED trial outcome (%)**	
** High**	139 (85.8)
** Low**	23 (14.2)
**Gleason grade group (%)**	
** 1**	11 (9.32)
** 2**	16 (13.56)
** 3**	16 (13.56)
** 4**	22 (18.03)
** 5**	53 (43.44)
**Previous taxane use (%)**	
** No**	32 (19.75)
** Yes**	130 (80.25)
**Patient age**	
** Total *N***	163
** Mean (std dev)**	72.56 (9.69)
** Median (Q1-Q3)**	73 (65-80)
** Min-max**	47-98
**Number of prior lines of therapy**	
** Total *N***	160
** Mean (SD)**	4.72 (1.75)
** Median (Q1-Q3)**	4 (3-6)
** Min-Max**	2-10
**Number of lutetium-177(^177^Lu)–PSMA-617 Cycles**	
** Total *N***	163
** Mean (SD)**	3.71 (1.84)
** Median (Q1-Q3)**	4 (2-6)
** Min-Max**	1-6
**Baseline ALP**	
** Total *N***	156
** Mean (SD)**	163.22 (169.63)
** Median (Q1-Q3)**	102.5 (72-177.5)
** Min-Max**	30-1299
**Baseline PSA**	
** Total *N***	155
** Mean (SD)**	254.14 (372.78)
** Median (Q1-Q3)**	68.46 (20.44-342)
** Min-Max**	0.1-1300
**Body mass index**	
** Total *N***	161
** Mean (SD)**	27.74 (5.3)
** Median (Q1-Q3)**	26.47 (23.43-30.88)
** Min-Max**	18.05-44.8
**Change in BMI from start to end of treatment**	
** Total *N***	147
** Mean (SD)**	1.4 (2.85)
** Median (Q1-Q3)**	0.97 (0-2.57)
** Min-Max**	−5.16-23.3
**Psoas muscle index**	
** Total *N***	155
** Mean (SD)**	6.12 (1.66)
** Median (Q1-Q3)**	6.05 (4.82-7.09)
** Min-Max**	2.55-10.8
**Skeletal muscle index**	
** Total *N***	153
** Mean (SD)**	41.83 (7.75)
** Median (Q1-Q3)**	41.21 (36.27-47.38)
** Min-Max**	24.93-66.48
**Visceral adipose tissue index**	
** Total *N***	157
** Mean (SD)**	58.74 (34.48)
** Median (Q1-Q3)**	55.96 (32.53-77.36)
** Min-Max**	9.99-210.1
**Subcutaneous adipose tissue index**	
** Total *N***	155
** Mean (SD)**	74.09 (38.44)
** Median (Q1-Q3)**	66.63 (48.04-94.55)
** Min-Max**	21.0-205.34
**Total adipose tissue index**	
** Total *N***	155
** Mean (SD)**	131.55 (64.25)
** Median (Q1-Q3)**	123.76 (84.92-171.17)
** Min-Max**	38.79-312.75
** *N* missing**	8
**Intermuscular fat index**	
** Total *N***	156
** Mean (SD)**	4.54 (2.93)
** Median (Q1-Q3)**	3.93 (2.53-5.77)
** Min-Max**	0.57-17.61
** *N* missing**	7
**Myosteatosis**	
** Total *N***	152
** Mean (SD)**	11.67 (7.68)
** Median (Q1-Q3)**	9.76 (5.86-15.45)
** Min-Max**	1.47-47.05
** *N* missing**	11
**Baseline body mass index, *N* (%)**	
** <25**	61 (37.42)
** ≥25 and ≤30**	55 (33.74)
** >30**	55 (33.74)
**Change in body mass index during treatment, *N* (%)**	
** Decrease**	107 (74.83)
** Increase**	36 (25.17)
**Psoas muscle index (PMI) (%)**	
** ≥4.7**	122 (75.31)
** <4.7**	40 (24.69)
**Skeletal muscle index (SMI), *N* (%)**	
** ≥47.4**	37 (22.7)
** <47.4**	126 (77.3)
**Visceral adipose tissue index (VATI), *N* (%)**	
** ≥38.5**	106 (65.03)
** <38.5**	57 (34.97)
**Subcutaneous adipose tissue index (SATI), *N* (%)**	
** ≥72.2**	66 (40.49)
** <72.2**	97 (59.51)
**Total adipose tissue index (TATI), *N* (%)**	
** ≥171.2**	38 (23.31)
** <171.2**	125 (76.69)
**Visceral fat-to-subcutaneous fat ratio (VATI/SATI) (%)**	
** ≥0.64**	97 (59.51)
** <0.64**	66 (40.49)
**Subcutaneous fat-to-muscle ratio (SATI/SMI) (%)**	
** ≥1.62**	75 (46.01)
** <1.62**	88 (53.99)
**Intermuscular fat index (IFI) (%)**	
** ≥4.87**	56 (34.36)
** <4.87**	107 (65.64)

### Body composition metrics

**Figure 1.**
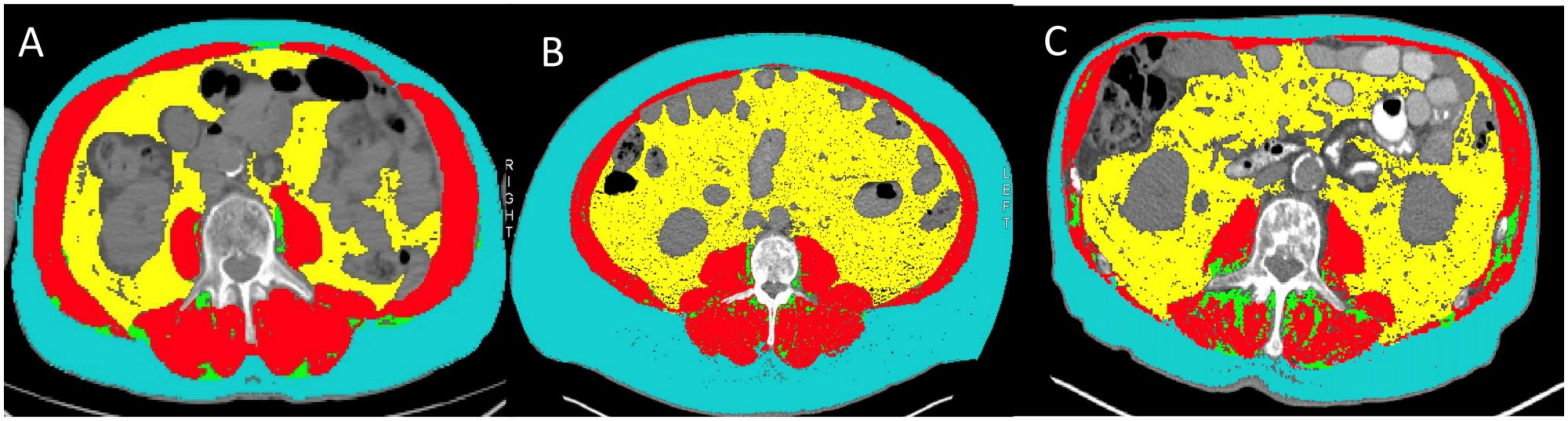
(A-C) Segmentation results from a CT-derived body composition metrics using Slice-O-matic. (A) Patient with OS 1 month and 15 days with BMI 23.4, SATI/SMI: 0.99; VATI/SATI. (B) Patient with OS 11 Months and 7 days with BMI 39.95, SATI/SMI: 2.76, VATI/SATI: 0.65. (C) Patient with OS 5 month and 4 days with BMI: 24, SATI/SMI: 1.31, VATI/SATI: 1.41

Representative examples of L3 segmentation across varying BMI measurements and body composition metrics are displayed in Figure 1A-C. The results of univariate analyses evaluating associations between body composition metrics and OS, PFS, and PSA50 response are presented in Tables S1-S3. The results of the multivariable analyses evaluating body composition metrics in relation to PSA50 response, PFS, and OS are shown in Table 2. Each body composition metric was evaluated in an independent multivariable model separate from other body composition metrics and adjusted for age, race, previous taxane use, number of lines of prior therapy, and baseline PSA. Compared with patients with a BMI ≥ 30, patients with a BMI < 25 had lower odds of achieving a PSA50 response (odds ratio [OR] = 0.28, 95% confidence interval [CI], 0.10-0.78, *P* = .015), and patients with a BMI of 25 to <30 demonstrated a nonsignificant trend toward lower odds of a PSA50 response (OR = 0.45, 95% CI, 0.17-1.16, *P* = .099). The results of univariable and multivariable analysis of body composition metrics using continuous data without dichotomization are presented in Tables S4 and S5.

**Table 2.** Multivariable analysis of body composition metrics and PSA50, PFS, and OS.^a^

Body composition metric	Cutoff	PSA50 odds ratio (95% CI), *P*	PFS hazard ratio (95% CI), *P*	OS hazard ratio (95% CI), *P*
**Baseline body mass index**	<25	0.28 (0.10-0.78) *P* = .015^a^	1.15 (0.69-1.93) *P* = .592	1.98 (0.94-4.17) *P* = .073
	≥25 and <30	0.45 (0.17-1.16) *P* = .099	1.05 (0.64-1.72) *P* = .854	1.49 (0.7-3.17) *P* = .3
**Subcutaneous adipose tissue index/skeletal muscle index**	≥1.62	1.40 (0.68-2.88) *P* = .356	0.87 (0.58-1.30) *P* = .493	0.50 (0.28-0.89) *P* = .019^a^
**Visceral adipose tissue index/subcutaneous adipose tissue index**	≥0.64	0.78 (0.38-1.64) *P* = .519	1.36 (0.89-2.07) *P* = .157	2.62 (1.34-5.11) *P* = .005^a^
**Visceral adipose tissue index**	≥38.5	0.81 (0.38-1.71) *P* = .58	1.54 (1.00-2.39) *P* = .052	1.42 (0.77-2.63) *P* = .263
**Subcutaneous adipose tissue index**	≥72	1.45 (0.68-3.09), *P* = .333	0.87 (0.58-1.30) *P* = .493	0.57 (0.32-1.02) *P* = .06
**Skeletal muscle index**	≥47.4	2.59 (0.97-6.91), *P* = .058	0.71 (0.42-1.21) *P* = .212	0.51 (0.23-1.13) *P* = .097
**Body mass index decline**	Decrease	0.72 (0.31-1.68) *P* = .45	1.75 (1.06-2.91) *P* = .03^a^	2.35 (1.08-5.09) *P* = .031^a^
**Total adipose tissue index**	≥171.2	2.12 (0.86-5.21), *P* = .103	0.89 (0.56-1.42), *P* = .621	0.65 (0.34-1.26) *P* = .204
**Intermuscular fat index**	≥4.87	1.30 (0.61-2.78) *P* = .502	0.82 (0.53-1.27) *P* = .378	0.70 (0.38-1.28) *P* = .244
**Myosteatosis**	≥4.14	0.63 (0.24-1.66) *P* = .35	1.65 (0.94-2.89) *P* = .081	1.87 (0.84-4.16) *P* = .126
**Psoas muscle index**	≥4.7	0.90 (0.37-2.14) *P* = .806	0.93 (0.59-1.49) *P* = .774	0.68 (0.37-1.26) *P* = .217

a Portions of this data was presented at GU ASCO 2026 annual meeting.

For PFS, a decline in BMI during treatment with ^177^Lu–PSMA-617 was associated with shorter PFS (HR = 1.75, 95% CI, 1.06-2.91, *P* = .03). Higher VATI displayed a nonsignificant trend toward shorter PFS on multivariable analysis (HR = 1.54, 95% CI, 1.00-2.39, *P* = .052). No additional body composition metrics reached statistical significance in association with PFS on multivariable analyses.

**Figure 2.**
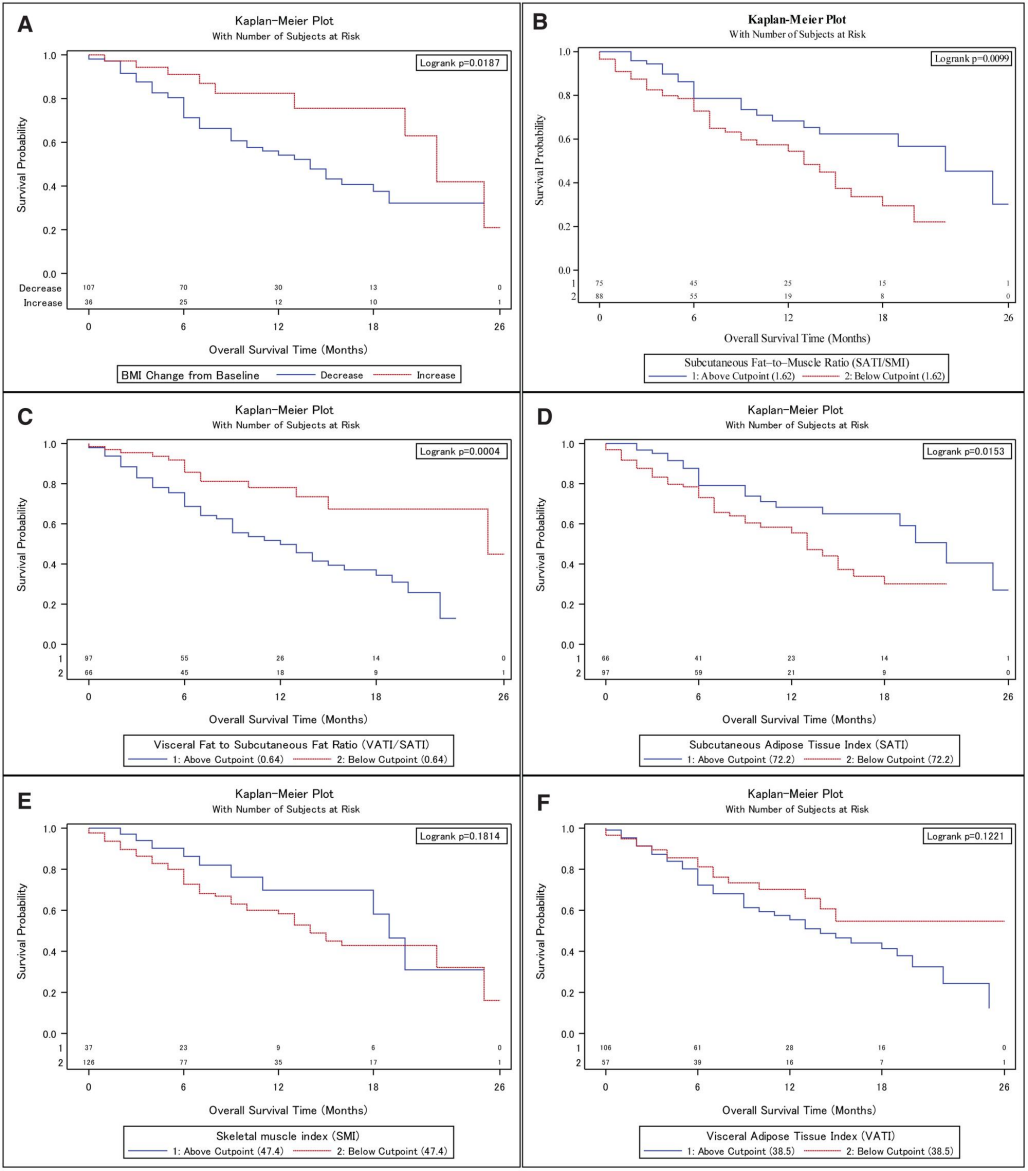
(A-F) Kaplan–Meier plot of computerized-tomography-based body composition metrics and overall survival. (A) Kaplan–Meier plot of body mass index change from the initiation of ^177^Lu–PSMA-617 to end of treatment. (B) Kaplan–Meier plot of overall survival stratified by subcutaneous fat-to-muscle ratio (≥1.62 vs <1.62). (C) Kaplan–Meier plot of overall survival stratified by visceral fat-to-subcutaneous fat ratio (≥0.64 vs <0.64). (D) Kaplan–Meier plot of overall survival stratified by subcutaneous adipose tissue index (≥72.2 vs <72.2). (E) Kaplan–Meier plot of overall survival stratified by skeletal muscle index (≥47.4 vs <47.4). (F) Kaplan–Meier plot of overall survival stratified by visceral adipose tissue index (≥38.5 vs < 38.5).

For OS, a decline in BMI during treatment was also associated with shorter OS (HR = 2.35, 95% CI, 1.08-5.09, *P* = .031). Higher VATI/SATI was associated with shorter OS (HR = 2.62, 95% CI, 1.34-5.11, *P* = .005). Higher SATI/SMI was associated with improved OS (HR = 0.5, 95% CI, 0.28-0.89, *P* = .019). Higher SATI and SMI displayed a nonsignificant association with improved OS (SATI HR = 0.57, 95% CI, 0.32-1.02, *P* = .06; SMI HR = 0.51, 95% CI, 0.23-1.13, *P* = .097).^21^ Kaplan–Meier plots of overall survival stratified by body composition metrics are presented in Figure 2A-F.

### Novel risk scoring system

A prognostic model was constructed using best-subset variable selection from baseline patient characteristics. Among the candidate variables, Gleason grade, number of prior lines of therapy, ECOG performance status, and age had the greatest contribution to model performance, yielding a C-index of 0.6961, as displayed in Table S6.

A separate prognostic model based on body composition metrics was developed from the candidate variables of BMI, PMI, SMI, VATI, SATI, TATI, VATI/SATI, SATI/SMI, IFI, myosteatosis, and skeletal muscle attenuation (mean Hounsfield units). VATI/SATI (*P* = .0006), SMI, and TATI emerged as the strongest prognostic indicators in model performance, as displayed in Table 3.

**Table 3.** Prognostic model based on body composition metrics.

Body composition metrics model, *N* = 158			
Predictor	Estimate	95% CI	*P*-value
**Visceral fat-to-subcutaneous fat ratio**	0.931	0.398 to 1.46	**.0006** ^*^
**Skeletal muscle index**	−0.00380	−0.0408 to 0.0332	.841
**Total adipose tissue index**	−0.00384	−0.00876 to 0.00108	.126

Score_OS = _0.931×*VATI/SATI*—0.0038×*SMI*—0.00384×*TATI*.

Model Performance: C-index: 0.629.

* Independently associated with overall survival.

We applied a previously published prognostic biomarker score using blood-based inflammatory markers, Score = −0.00078×NER−0.00011×PLR+0.00038×SII−0.42×Hgb, which was re-estimated in the current cohort.^7^ In the present cohort, the biomarker model had a C-index of 0.701, as evaluated using the cohort of 158 patients who had complete body composition data.

A cumulative prognostic model was then constructed by integrating candidate variables from the clinical characteristics, body composition, and biomarker prognostic models. VATI/SATI, ECOG performance status, PLR, NER, SII, and hemoglobin (*P* ≤ .0001) emerged as prognostically significant in the composite model as displayed in Table 4. The composite prognostic model had a C-index of 0.732. This is a significant improvement in prognostic performance compared to the baseline patient characteristic model (*P* < .0001) and the body composition score (*P* < .0001), when analyzed using the CompareC package. However, no significant difference was observed between the composite model and the previously published biomarker model (*P* = .200).

**Table 4.** Composite prognostic risk scoring system.

Composite prognostic risk score, *N* = 158			
Predictor	Estimate	95% CI	*P*-value
**VATI/SATI**	0.456	−0.153 to 1.066	.142
**ECOG performance status**	−0.000406	−0.00368 to 0.00287	.808
**Platelet-to-lymphocyte ratio**	−0.00013	−0.000358 to 0.00332	.939
**Neutrophil-to-eosinophil ratio (NER)**	−0.00103	−0.00395 to 0.00089	.294
**Systemic immune inflammation value**	0.000332	−0.0000303 to 0.000694	.0726
**Baseline hemoglobin**	−0.400	−0.589 to −0.212	**<.0001** ^*^

Score_OS_ = 0.456×V/S−0.000406×ECOG−0.000134×PLR−0.00103×NER+0.000332×SII−0.400×Hgb.

Model performance: −C-index: 0.732.

* Independently associated with overall survival.

**Figure 3.**
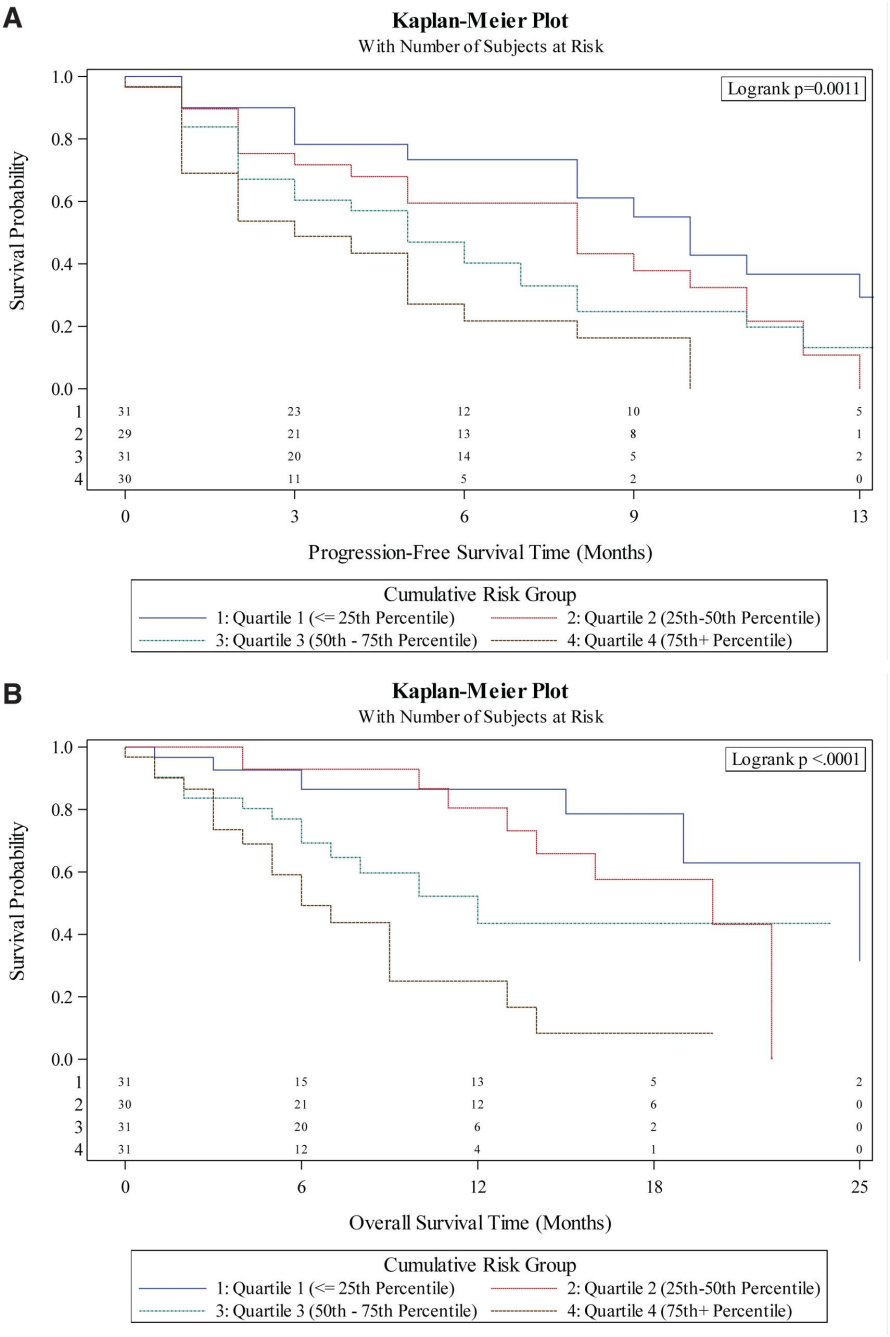
(A-B) Kaplan–Meier plot of PFS and OS by novel risk composite score. (A) Kaplan–Meier plot of progression-free survival when the novel composite risk score divides patients into quartiles from 1 (lowest risk) to 4 (highest risk). (B) Kaplan–Meier Plot of OS when the novel composite risk score to stratifies patients into quartiles from 1 to 4.

Patients were divided into quartiles based on the composite risk score, with quartile 1 (Q1) being the lowest risk group and quartile 4 (Q4) being the highest risk group. On multivariable analysis, stratification with the composite risk score was significantly associated with PFS (type 3 *P* = .034). Each quartile had a progressively reduced risk of progression compared to Q4 (Q1 HR = 0.34, 95% CI, 0.16-0.74, *P* = .007; Q2 HR = 0.40, 95% CI, 0.18-0.88, *P* = .022; Q3 HR = 0.62, 95% CI, 0.32-1.19, *P* = .149). Kaplan–Meier plots of PFS are presented in Figure 3A and demonstrated significant stratification (*P* = .0011). Patients in Q1 had a median PFS of 10 months (95% CI, 8, NA), Q2 had a median PFS of 8 months (95% CI, 4-11), Q3 had a median PFS of 5 months (95% CI, 2-7), and Q4 had a median PFS of 3 months (95% CI, 1-5).

The composite risk score also significantly stratified patients based on OS (multivariate analysis type 3 *P* = .003). Compared to Q4, patients in Q1 had an 85% reduced risk of death (HR = 0.15, 95% CI, 0.05-0.49, *P* = .002), patients in Q2 had an 81% reduced risk of death (HR = 0.19, 95% CI, 0.07-0.56, *P* = .003), and patients in Q3 had a 46% reduction in risk of death (HR = 0.54, 95% CI, 0.24-1.21, *P* = .133). Kaplan–Meier plots demonstrated significant stratification by OS (*P* < .0001) and are shown in Figure 3B.

## Discussion

We found that body composition metrics, including higher VATI/SATI, lower SATI/SMI, and a decline in BMI during treatment with ^177^Lu–PSMA-617 were independently associated with poorer survival outcomes for patients with mCRPC. Building on the established associations between body composition and systemic inflammation, we expanded upon previous research to develop a novel risk score incorporating body composition metrics. Within the composite prognostic model, SII, VATI/SATI, ECOG performance status, PLR, NER, and baseline hemoglobin were the highest contributors to overall model discrimination. The composite prognostic model demonstrated improved discriminative ability compared with clinical characteristics alone (C = 0.732 vs C = 0.6961, *P* < .0001), supporting its utility for risk stratification in patients considered for treatment with ^177^Lu–PSMA-617.

Body composition metrics have prognostic value in several malignancies, including prostate cancer.^21^ While higher BMI has paradoxically been associated with improved outcomes in mCRPC, current literature suggests that individual body composition metrics may be more informative than BMI alone.^11^^,^  ^22^ A large meta-analysis of men with prostate cancer at any treatment stage showed that higher subcutaneous adiposity, a lower visceral-to-subcutaneous fat ratio, and greater muscle mass were associated with improved survival.^23^ In patients with mCRPC treated with abiraterone or enzalutamide, higher subcutaneous fat was associated with improved survival.^11^ These metrics are especially relevant for patients with mCRPC. Patients often undergo prolonged androgen-deprivation therapy (ADT), which is associated with muscle loss, increased adiposity, and an average weight gain of 2.2 kg within the first year.^24^^,^^25^ Novel anti-hormonal therapies may further increase adiposity, with abiraterone and prednisone specifically associated with increased visceral adipose tissue.^26^^,^^27^ Consistent with this, our cohort of heavily pretreated men with mCRPC had a median SMI of 41.2 cm^2^/m^2^, which is lower than the commonly reported sarcopenia cutoff of 52-55 cm^2^/m^2^ for men, suggesting a lower threshold may be appropriate in this population. We hypothesize this reflects the cumulative effects of long-term anti-hormonal therapies.^28^^,^^29^

At present, limited data exist regarding the relationship between CT-derived body composition metrics and outcomes in patients treated with ^177^Lu–PSMA-617. Prior studies have included smaller sample sizes or use [177Lu]Lu-PSMA I&T, which is not approved in the United States.^30^^,^^31^ In the context of ^177^Lu–PSMA-617, lower BMI has been associated with early treatment discontinuation, progression, reduced PSA response rates, and shortened OS.^30^^,^  ^32^ Hamtrampf et al. and Kaplan et al. did not find consistent associations between individual body composition metrics and clinical outcomes.^30^^,^^31^ Our findings extend this literature by suggesting that ratio-based metrics may have greater prognostic significance than individual metrics alone. Our results show the significance of VATI/SATI and SATI/SMI ratios, compared to individual body composition metrics and BMI, and suggest that relative composition may be more clinically informative and should be included as a studied metric.

Given the potential limitations of derived dichotomized cutoff values in the analysis of body composition metrics, we also evaluated body composition metrics as continuous variables. These analyses were broadly consistent with the primary dichotomized findings. VATI/SATI remained independently significantly associated with OS when modeled continuously (HR = 2.07, 95% CI, 1.19-3.62, *P* = .011) and the SATI/SMI ratio showed a directionally similar association but did not meet statistical significance (HR = 0.69, 95% CI, 0.46-1.04, *P* = .074). Taken together, these findings support the prognostic relevance of relative body composition metrics and are consistent with emerging literature supporting the VATI/SATI ratio as an important prognostic indicator in other malignancies.^33^

A prognostic model integrating only clinical variables demonstrated limited discriminative ability (C = 0.6961), highlighting the potential utility of biomarkers. Given that patients considering treatment with ^177^Lu-PSMA-617 undergo a PSMA-PET-CT to determine eligibility, pretreatment CT-based body composition metrics provide a reliable and scalable approach to prognostication. A previously developed model based on blood-based inflammatory biomarkers also demonstrated discriminative ability (C-index = 0.702). Given the biological associations between visceral fat and systemic inflammation, we developed a composite prognostic score incorporating body composition metrics and inflammatory biomarkers.^34^ VATI/SATI, ECOG status, PLR, NER, SII, and baseline hemoglobin emerged as the best predictors of the overall survival composite score. The composite prognostic score has a C-index of 0.732, indicating strong discriminatory ability. The composite score C-index of 0.731 displayed statistically significant improvement in prognostication compared with the clinical variables alone (*P* < .001). However, the magnitude of improvement should be interpreted in the context of the additional complexity from obtaining body composition metrics, inflammatory biomarkers, and applying the novel composite score. The composite score needs external validation and investigation to determine if this incremental increase in prognostication is clinically meaningful and significant enough to account for the added complexity of applying the score. The clinical value of the composite score is highest when risk stratification is particularly beneficial, such as for clinical trial stratification or during patient counseling. The composite risk score can be used as a support tool to risk-stratify patients who are being considered or currently being treated with ^177^Lu-PSMA-617 and to help clinicians communicate expected prognosis. The composite score may also be clinically for useful to identify patients who may benefit from closer monitoring or early supportive care involvement. Prospective external validation is required to determine if the score has predictive value in selecting ^177^Lu-PSMA-617 over alternative therapeutic approaches.

There are several clinical considerations resulting from this study. In our cohort of patients with mCRPC and heavy pretreatment with anti-hormonal therapies, we found that a disproportionate percentage of patients were sarcopenic by standard criteria. Our findings suggest the importance of a multidisciplinary approach, including nutrition and physical therapy, to help patients to maintain muscle mass and reduce visceral adiposity. Our novel composite risk score incorporating body composition analysis can be used by oncologists to help inform clinical decision-making for patients who are considering treatment with ^177^Lu–PSMA-617. Furthermore, our risk score can be used as a tool to guide discussions when counseling patients and approaching conversations regarding expected outcomes. The composite score may also aid in risk stratification for clinical trials.

This study is hypothesis-generating and subject to several limitations. Our analysis is retrospective, which introduces the potential for selection bias. To mitigate this risk, we included all patients who received at least 1 cycle of treatment with ^177^Lu–PSMA-617 at Emory Winship Cancer Institute from July 10, 2022 to June 17, 2025. Unmeasured confounding factors may have influenced our results, including the variability of duration of ADT treatment among patients, which can alter body composition metrics. We aimed to minimize additional confounding variables by controlling for patient age, race, previous taxane use, number of prior lines of therapy, and baseline PSA in our analysis. CT-based body composition metrics were segmented semi-automatically, and the manual components of segmentation may introduce measurement error. To reduce interobserver variability, all segmentations were performed by 1 trained author (M.B.G.). To improve clinical interpretability, body composition cutoff values were dichotomized and derived from our cohort, which may limit generalizability. To address this limitation, we evaluated these metrics as continuous variables, yielding overall consistent findings. However, external validation in larger prospective cohorts is needed to determine the clinical utility of these thresholds. Future studies should collect detailed data on radiotherapy exposure, including treatment directed to the prostate and metastatic lesions, to determine if these therapies are associated with patient outcomes and the novel prognostic score. Prospective studies are needed to validate our findings and the novel composite risk score.

## Supplementary Material

oyag269_Supplementary_Data

## Data Availability

The data underlying this article will be shared on reasonable request to the corresponding author.

## References

[1] Sartor O , Bono J, Chi KN, et al; VISION Investigators. Lutetium-177–PSMA-617 for metastatic castration-resistant prostate cancer. N Engl J Med. 2021;385:1091-1103. :10.1056/NEJMoa2107322 34161051 PMC8446332

[2] Kratochwil C , Fendler WP, Eiber M, et al Joint EANM/SNMMI procedure guideline for the use of (177)Lu-labeled PSMA-targeted radioligand-therapy ((177)Lu-PSMA-RLT). Eur J Nucl Med Mol Imaging. 2023;50:2830-2845. 10.1007/s00259-023-06255-8 37246997 PMC10317889

[3] Gerke MB , Bedmutha A, Marra A, et al Clinical outcomes of lutetium-177-PSMA-617 in a racially diverse cohort of patients with metastatic castration-resistant prostate cancer. Oncol. 2026;31:oyag022. 10.1093/oncolo/oyag022 PMC12952921 41630499

[4] Gafita A , Calais J, Grogan TR, et al Nomograms to predict outcomes after ^177^Lu-PSMA therapy in men with metastatic castration-resistant prostate cancer: an international, multicentre, retrospective study. Lancet Oncol. 2021;22:1115-1125. 10.1016/S1470-2045(21)00274-6 34246328

[5] Herrmann K , Gafita A, de Bono JS, et al Multivariable models of outcomes with [^177^Lu]Lu-PSMA-617: analysis of the phase 3 VISION trial. EClinicalMedicine. 2024;77:102862. 10.1016/j.eclinm.2024.102862 39430616 PMC11490806

[6] Gerke M , Marra A, Liu Y, et al Real world outcomes of ^177^Lu-PSMA-617 PSMA in a racially diverse cohort of patients with metastatic castration resistant prostate cancer (mCRPC). J Clin Oncol. 2025;43:97-97. 10.1200/JCO.2025.43.5_suppl.97

[7] Gerke MB , Marra A, Liu Y, et al Inflammatory biomarkers have a prognostic role in patients with metastatic castration-resistant prostate cancer treated with lutetium-177-PSMA-617. Cancer. 2026;132:e70410. 10.1002/cncr.70410 41989013

[8] Yu JY , Choi WJ, Lee HS, Lee JW. Relationship between inflammatory markers and visceral obesity in obese and overweight korean adults: an observational study. Medicine (Baltimore). 2019;98:e14740. 10.1097/md.0000000000014740 30817629 PMC6831265

[9] Bilen MA , Martini DJ, Liu Y, et al Combined effect of sarcopenia and systemic inflammation on survival in patients with advanced stage cancer treated with immunotherapy. Oncologist. 2020;25:e528-e535. 10.1634/theoncologist.2019-0751 32162807 PMC7066707

[10] Schaap LA , Pluijm SM, Deeg DJ, Visser M. Inflammatory markers and loss of muscle mass (sarcopenia) and strength. Am J Med. 2006;119:526.e9-17. 10.1016/j.amjmed.2005.10.049 16750969

[11] Antoun S , Bayar A, Ileana E, et al High subcutaneous adipose tissue predicts the prognosis in metastatic castration-resistant prostate cancer patients in post chemotherapy setting. Eur J Cancer. 2015;51:2570-2577. 10.1016/j.ejca.2015.07.042 26278649

[12] Finley DS , Calvert VS, Inokuchi J, et al Periprostatic adipose tissue as a modulator of prostate cancer aggressiveness. J Urol. 2009;182:1621-1627. 10.1016/j.juro.2009.06.015 19683746

[13] Ibrahim MM. Subcutaneous and visceral adipose tissue: structural and functional differences. Obes Rev. 2010;11:11-18. 10.1111/j.1467-789X.2009.00623.x 19656312

[14] National Cancer Institute . *Common Terminology Criteria for Adverse Events (CTCAE)*. Version 6.0. Cancer Therapy Evaluation Program, Division of Cancer Therapy and Diagnosis; 2025. https://dctd.cancer.gov/research/ctep-trials/for-sites/adverse-events

[15] Martini DJ , Olsen TA, Goyal S, et al Body composition variables as radiographic biomarkers of clinical outcomes in metastatic renal cell carcinoma patients receiving immune checkpoint inhibitors. Original research. Front Oncol. 2021;11:707050. 10.3389/fonc.2021.707050 34307176 PMC8299332

[16] Mitsiopoulos N , Baumgartner RN, Heymsfield SB, Lyons W, Gallagher D, Ross R. Cadaver validation of skeletal muscle measurement by magnetic resonance imaging and computerized tomography. J Appl Physiol. 1998;85:115-122. 10.1152/jappl.1998.85.1.115 9655763

[17] Martini DJ , Shabto JM, Goyal S, et al Body composition as an independent predictive and prognostic biomarker in advanced urothelial carcinoma patients treated with immune checkpoint inhibitors. Oncologist. 2021;26:1017-1025. 10.1002/onco.13922 34342095 PMC8649001

[18] Liu Y , Nickleach DC, Zhang C, Switchenko JM, Kowalski J. Carrying out streamlined routine data analyses with reports for observational studies: introduction to a series of generic SAS® macros. F1000Res. 2019;7:1955. 10.12688/f1000research.16866.1 PMC6567291 31231506

[19] Scher HI , Morris MJ, Stadler WM, et al; Prostate Cancer Clinical Trials Working Group 3. Trial design and objectives for castration-resistant prostate cancer: updated recommendations from the prostate cancer clinical trials working group 3. J Clin Oncol. 2016;34:1402-1418. 10.1200/jco.2015.64.2702 26903579 PMC4872347

[20] Kang L , Chen W, Petrick NA, Gallas BD. Comparing two correlated C indices with right-censored survival outcome: a one-shot nonparametric approach. Stat Med. 2015;34:685-703. 10.1002/sim.6370 25399736 PMC4314453

[21] Gerke M , Marra A, Liu Y, et al Body composition metrics and clinical outcomes for patients with metastatic castration-resistant prostate cancer (mCRPC) treated with lutetium-177–PSMA-617. J Clin Oncol. 2026;44:241-241. 10.1200/JCO.2026.44.7_suppl.241

[22] Halabi S , Ou SS, Vogelzang NJ, Small EJ. Inverse correlation between body mass index and clinical outcomes in men with advanced castration-recurrent prostate cancer. Cancer. 2007;110:1478-1484. 10.1002/cncr.22932 17665494

[23] Lopez P , Newton RU, Taaffe DR, et al Associations of fat and muscle mass with overall survival in men with prostate cancer: a systematic review with meta-analysis. Prostate Cancer Prostatic Dis. 2022;25:615-626. 10.1038/s41391-021-00442-0 34420038 PMC9705235

[24] Smith MR. Changes in fat and lean body mass during androgen-deprivation therapy for prostate cancer. Urology. 2004;63:742-745. 10.1016/j.urology.2003.10.063 15072892

[25] Kim HS , Moreira DM, Smith MR, et al A natural history of weight change in men with prostate cancer on androgen-deprivation therapy (ADT): results from the shared equal access regional cancer hospital (SEARCH) database. BJU Int. 2011;107:924-928. 10.1111/j.1464-410X.2010.09679.x 20860651 PMC3055926

[26] Buffoni M , Dalla Volta A, Valcamonico F, et al Total and regional changes in body composition in metastatic hormone-sensitive prostate cancer patients randomized to receive androgen deprivation + enzalutamide ± zoledronic acid. The BONENZA study. Eur Urol Oncol. 2025;8:782-791. 10.1016/j.euo.2025.02.006 40300921

[27] Blow TA , Murthy A, Grover R, et al Profiling of skeletal muscle and adipose tissue depots in men with advanced prostate cancer receiving different forms of androgen deprivation therapy. Eur Urol Open Sci. 2023;57:1-7. 10.1016/j.euros.2023.09.004 38020528 PMC10658404

[28] Tagliafico AS , Bignotti B, Torri L, Rossi F. Sarcopenia: how to measure, when and why. Radiol Med. 2022;127:228-237. 10.1007/s11547-022-01450-3 35041137 PMC8960583

[29] Amini B , Boyle SP, Boutin RD, Lenchik L. Approaches to assessment of muscle mass and myosteatosis on computed tomography: a systematic review. J Gerontol A Biol Sci Med Sci. 2019;74:1671-1678. 10.1093/gerona/glz034 30726878 PMC7357454

[30] Hartrampf PE , Mihatsch PW, Seitz AK, et al Elevated body mass index is associated with improved overall survival in castration-resistant prostate cancer patients undergoing Prostate-Specific membrane antigen-directed radioligand therapy. Journal of Nuclear Medicine. 2023;64:1272-1278. 10.2967/jnumed.122.265379 37290794

[31] Kaplan İ , Kömek H, Can C, et al Effect of body composition parameters, including sarcopenia, myosteatosis, and adipose tissue, on overall survival of patients with mCRPC receiving 177Lu-PSMA PRRT. Nucl Med Commun. 2025;46:592-598. 10.1097/mnm.0000000000001985 40308059

[32] Peslier H , Seegers V, Dufour PA. Study of predictive factors for response to (177)LU-PSMA in patients with metastatic castration-resistant prostate cancer. Front Med (Lausanne). 2025;12:1538507. 10.3389/fmed.2025.1538507 40166057 PMC11955661

[33] Wada M , Yamaguchi K, Yamakage H, et al Visceral-to-subcutaneous fat ratio is a possible prognostic factor for type 1 endometrial cancer. Int J Clin Oncol. 2022;27:434-440. 10.1007/s10147-021-02060-1 34716844 PMC8816789

[34] Fain JN , Madan AK, Hiler ML, Cheema P, Bahouth SW. Comparison of the release of adipokines by adipose tissue, adipose tissue matrix, and adipocytes from visceral and subcutaneous abdominal adipose tissues of obese humans. Endocrinology. 2004;145:2273-2282. 10.1210/en.2003-1336 14726444

