# Supplementary material for: Novel risk scoring system for patients with metastatic castration-resistant prostate cancer treated with lutetium-177–PSMA-617
Source: Oncologist. 2026 Jul 11;31(8):oyag269. doi: 10.1093/oncolo/oyag269 (PMC13395084; doi:10.1093/oncolo/oyag269)
Supplement: oyag269_Supplementary_Data [file oyag269_supplementary_data.zip › Supplementary Information.docx]

**Supplementary Table S1. Univariate Analysis Body Composition Variables and Overall Survival**

|  | | | **Overall Survival Time (Months)** | | |
| --- | --- | --- | --- | --- | --- |
| **Covariate** | **Level** | **N** | **Hazard Ratio (95% CI)** | **HR P-value** | **Log-rank P-value** |
| BMI Change from Baseline | Increase | 36 | 0.45 (0.21-0.88) | **0.030** | **0.019** |
|  | Decrease | 107 | - | **-** |  |
| Baseline BMI | <25 | 61 | 1.99 (1.03-4.13) | 0.052 | 0.117 |
|  | ≥25 and <30 | 55 | 1.65 (0.83-3.46) | 0.172 |  |
|  | ≥30 | 47 | - | **-** |  |
| Psoas Muscle Index | ≥4.7 | 122 | 0.71 (0.41-1.26) | 0.224 | 0.240 |
|  | <4.7 | 40 | - | **-** |  |
| Skeletal muscle index | >47.4 | 37 | 0.66 (0.32-1.23) | 0.228 | 0.181 |
|  | <47.4 | 126 | - | **-** |  |
| Visceral Adipose Tissue Index | ≥38.5 | 106 | 1.51 (0.89-2.69) | 0.146 | 0.122 |
|  | <38.5 | 57 | - | **-** |  |
| Subcutaneous Adipose Tissue Index | ≥72.2 | 66 | 0.52 (0.30-0.89) | **0.021** | **0.015** |
|  | <72.2 | 97 | - | **-** |  |
| Total Adipose Tissue Index | ≥171.2 | 38 | 0.70 (0.36-1.26) | 0.265 | 0.220 |
|  | <171.2 | 125 | - | **-** |  |
| Visceral Fat to Subcutaneous Fat Ratio | ≥0.64 | 97 | 2.76 (1.55-5.26) | **0.001** | **<.001** |
|  | <0.64 | 66 | - | **-** |  |
| Subcutaneous Fat to Muscle Ratio | ≥1.62 | 75 | 0.51 (0.30-0.86) | **0.014** | **0.010** |
|  | <1.62 | 88 | - | **-** |  |
| Intermuscular Fat Index (IFI) | ≥4.87 | 56 | 0.74 (0.41-1.27) | 0.298 | 0.260 |
|  | <4.87 | 107 | - | **-** |  |
| Myosteatosis percentage (IFI/SMI × 100%) | ≥4.14 | 135 | 1.56 (0.82-3.33) | 0.209 | 0.163 |
|  | <4.14 | 28 | - | **-** |  |
| Skeletal Muscle Average HU | ≥41.2 | 153 | 1.12 (0.44-4.07) | 0.838 | 0.657 |
|  | <41.2 | 10 | - | **-** |  |
| Previous Taxane Use | No | 26 | 0.98 (0.44-1.92) | 0.948 | 0.843 |
|  | Yes | 130 | - | **-** |  |
| Patient Race | White | 80 | 1.24 (0.73-2.14) | 0.427 | 0.706 |
|  | Other | 16 | 1.22 (0.50-2.65) | 0.648 |  |
|  | Black | 66 | - | **-** |  |

**Supplementary Table S2. Univariate Analysis of Body Composition Factors and Progression Free Survival**

|  | | | **Progression-Free Survival Time (Months)** | | |
| --- | --- | --- | --- | --- | --- |
| **Covariate** | **Level** | **N** | **Hazard Ratio (95% CI)** | **HR P-value** | **Log-rank P-value** |
| Body Mass Index (BMI) Change from Baseline | Increase | 36 | 0.61 (0.37-0.98) | **0.049** | **0.028** |
|  | Decrease | 107 | - | **-** |  |
| Baseline BMI | <25 | 60 | 1.09 (0.69-1.73) | 0.720 | 0.921 |
|  | ≥25 and <30 | 55 | 1.06 (0.67-1.68) | 0.814 |  |
|  | ≥30 | 47 | - | **-** |  |
| Psoas Muscle Index (PMI) | ≥4.7 | 121 | 1.04 (0.69-1.62) | 0.847 | 0.792 |
|  | <4.7 | 40 | - | **-** |  |
| Skeletal muscle index (SMI) | >47.4 | 36 | 0.91 (0.56-1.41) | 0.674 | 0.600 |
|  | <47.4 | 126 | - | **-** |  |
| Visceral Adipose Tissue Index (VATI) | ≥38.5 | 105 | 1.57 (1.07-2.37) | **0.027** | **0.015** |
|  | <38.5 | 57 | - | **-** |  |
| Subcutaneous Adipose Tissue Index (SATI) | ≥72.2 | 66 | 1.05 (0.72-1.52) | 0.803 | 0.800 |
|  | <72.2 | 96 | - | **-** |  |
| Total Adipose Tissue Index (TATI) | ≥171.2 | 38 | 0.93 (0.59-1.42) | 0.762 | 0.699 |
|  | <171.2 | 124 | - | **-** |  |
| Visceral Fat to Subcutaneous Fat Ratio (VATI/SATI) | ≥0.64 | 96 | 1.41 (0.97-2.09) | 0.076 | 0.053 |
|  | <0.64 | 66 | - | **-** |  |
| Subcutaneous Fat to Muscle Ratio (SATI/SMI) | ≥1.62 | 75 | 0.86 (0.59-1.25) | 0.440 | 0.403 |
|  | <1.62 | 87 | - | **-** |  |
| Intermuscular Fat Index (IFI) | ≥4.87 | 56 | 0.85 (0.57-1.26) | 0.436 | 0.383 |
|  | <4.87 | 106 | - | **-** |  |
| Myosteatosis percentage (IFI/SMI × 100%) | ≥4.14 | 134 | 1.49 (0.93-2.53) | 0.117 | 0.077 |
|  | <4.14 | 28 | - | **-** |  |
| Previous Taxane Use | No | 26 | 0.84 (0.47-1.40) | 0.540 | 0.451 |
|  | Yes | 129 | - | **-** |  |
| Patient Race | White | 80 | 1.03 (0.70-1.53) | 0.870 | 0.334 |
|  | Other | 16 | 1.57 (0.82-2.81) | 0.150 |  |
|  | Black | 65 | - | **-** |  |

**Supplementary Table S3. Univariate Analysis of Body Composition Factors and PSA50 Response**

|  | | | **PSA50** | |  |
| --- | --- | --- | --- | --- | --- |
| **Covariate** | **Statistics** | **Level** | **No N=67** | **Yes N=83** | **P-value*** |
| Number of Prior Lines of Therapy | N |  | 67 | 81 | 0.185 |
|  | Mean |  | 4.9 | 4.5 |  |
|  | Median |  | 5 | 4 |  |
| Baseline Body Mass Index | N (Col %) | <25 | 27 (40.3) | 27 (32.5) | 0.360 |
|  | N (Col %) | ≥25 and <30 | 15 (22.4) | 27 (32.5) |  |
|  | N (Col %) | ≥30 | 25 (37.3) | 29 (34.9) |  |
| Psoas Muscle Index (PMI) | N (Col %) | ≥4.7 | 52 (77.6) | 60 (73.2) | 0.532 |
|  | N (Col %) | <4.7 | 15 (22.4) | 22 (26.8) |  |
| Skeletal Muscle Index (SMI) | N (Col %) | ≥47.4 | 11 (16.4) | 19 (22.9) | 0.324 |
|  | N (Col %) | <47.4 | 56 (83.6) | 64 (77.1) |  |
| Visceral Adipose Tissue Index (VATI) | N (Col %) | ≥38.5 | 47 (70.1) | 50 (60.2) | 0.207 |
|  | N (Col %) | <38.5 | 20 (29.9) | 33 (39.8) |  |
| Subcutaneous Adipose Tissue Index (SATI) | N (Col %) | ≥72.2 | 26 (38.8) | 34 (41) | 0.789 |
|  | N (Col %) | <72.2 | 41 (61.2) | 49 (59) |  |
| Total Adipose Tissue Index (TATI) | N (Col %) | ≥171.2 | 13 (19.4) | 21 (25.3) | 0.391 |
|  | N (Col %) | <171.2 | 54 (80.6) | 62 (74.7) |  |
| Visceral Fat to Subcutaneous Fat Ratio (VATI/SATI) | N (Col %) | ≥0.64 | 43 (64.2) | 44 (53) | 0.168 |
|  | N (Col %) | <0.64 | 24 (35.8) | 39 (47) |  |
| Subcutaneous Fat to Muscle Ratio (SATI/SMI) | N (Col %) | ≥1.62 | 30 (44.8) | 40 (48.2) | 0.677 |
|  | N (Col %) | <1.62 | 37 (55.2) | 43 (51.8) |  |
| Intermuscular Fat Index (IFI) | N (Col %) | ≥4.87 | 22 (32.8) | 29 (34.9) | 0.787 |
|  | N (Col %) | <4.87 | 45 (67.2) | 54 (65.1) |  |
| Myosteatosis percentage (IFI/SMI × 100%) | N (Col %) | ≥4.14 | 57 (85.1) | 65 (78.3) | 0.291 |
|  | N (Col %) | <4.14 | 10 (14.9) | 18 (21.7) |  |
| Patient Race | N (Col %) | Black | 23 (34.3) | 40 (48.8) | 0.148 |
|  | N (Col %) | Other | 8 (11.9) | 5 (6.1) |  |
|  | N (Col %) | White | 36 (53.7) | 37 (45.1) |  |
| Patient Age | N |  | 67 | 83 | **0.041** |
|  | Mean |  | 70.7 | 73.9 |  |
|  | Median |  | 71 | 75 |  |
| Previous Taxane Use | N (Col %) | No | 7 (10.8) | 16 (20.5) | 0.114 |
|  | N (Col %) | Yes | 58 (89.2) | 62 (79.5) |  |
| Baseline PSA | N |  | 64 | 81 | **0.049** |
|  | Mean |  | 317.8 | 194.8 |  |
|  | Median |  | 93.4 | 45.3 |  |
| BMI Change from Baseline | N (Col %) | Decrease | 48 (78.7) | 54 (70.1) | 0.255 |
|  | N (Col %) | Increase | 13 (21.3) | 23 (29.9) |  |
| *  The p-value is calculated by ANOVA for numerical covariates; and chi-square test or Fisher's exact for categorical covariates, where appropriate. | | | | | |

**Supplementary Table S4. Continuous Univariable Analysis of Body Composition Variables**

|  | | **Overall Survival Time (Months)** | |
| --- | --- | --- | --- |
| **Covariate** | **N** | **Hazard Ratio (95% CI)** | **HR P-value** |
| Psoas Muscle Index | 155 | 0.98 (0.84-1.15) | 0.847 |
| Subcutaneous Adipose Tissue Index | 155 | 0.99 (0.98-1.00) | **0.041** |
| Subcutaneous Adipose Tissue Index/Skeletal Muscle Index | 150 | 0.68 (0.46-0.99) | 0.054 |
| Skeletal Muscle Index | 153 | 0.98 (0.95-1.02) | 0.337 |
| Total Adipose Tissue Index | 155 | 1.00 (0.99-1.00) | 0.492 |
| Visceral Adipose Tissue Index | 157 | 1.00 (0.99-1.01) | 0.651 |
| Visceral Adipose Tissue Index / Subcutaneous Adipose Tissue Index | 149 | 2.27 (1.34-3.72) | **0.002** |
| Firth’s penalized maximum likelihood estimation was used. | | | |

**Supplementary Table S5. Continuous Multivariable Analysis of Body Composition Variables**

| **Body Composition Metric** | **HR Scale** | **N** | **HR (95% CI)** | **P-value** |
| --- | --- | --- | --- | --- |
| Baseline Body Mass Index | 5 kg/m^2^ increase | 147 | 0.76 (0.57-1.03) | 0.081 |
| Subcutaneous Adipose Tissue Index/Skeletal Muscle Index | 1-unit increase | 136 | 0.69 (0.46-1.04) | 0.074 |
| Visceral Adipose Tissue Index/Subcutaneous Adipose Tissue Index | 1-unit increase | 135 | 2.07 (1.19-3.62) | **0.011** |
| Visceral Adipose Tissue Index | 10-unit increase | 141 | 1.01 (0.94-1.09) | 0.799 |
| Subcutaneous Adipose Tissue Index | 10-unit increase | 139 | 0.92 (0.85-1.01) | 0.076 |
| Skeletal Muscle Index | 5-unit increase | 139 | 0.91 (0.73-1.14) | 0.428 |
| Total Adipose Tissue Index | 10-unit increase | 139 | 0.99 (9.97-1.02) | 0.553 |
| Intermuscular Fat Index | 1-unit increase | 140 | 0.96 (0.86-1.06) | 0.383 |
| Myosteatosis | 5-unit increase | 138 | 0.96 (0.79-1.18) | 0.718 |
| Psoas Muscle Index | 1-unit increase | 141 | 0.96 (0.80-1.14) | 0.632 |

**Supplementary Table S6. Clinical Characteristics Prognostic Model**

| **Clinical Characteristics Model** |
| --- |
| \| Predictor \| Estimate \| 95% CI \| P-value \| \| --- \| --- \| --- \| --- \| \| Gleason Grade \| -0.18076 \| -0.3929 – 0.0389 \| 0.101 \| \| Prior Lines of Therapy \| 0.07710 \| -0.0846 – 0.2359 \| 0.347 \| \| Age \| -0.02835 \| -0.0617 – 0.0061 \| 0.102 \| \| ECOG Performance Status \| 0.96274 \| 0.4528 – 1.4228 \| **0.0001** \|   Score_OS_ = -0.181**Gleason*+ 0.0771**PriorLine* – 0.0284**Age*+ 0.963*ECOG |
| Model Performance: C-index: 0.6961 |
